# Supplementary material for: Large-gap insulating dimer ground state in monolayer IrTe2
Source: Nat Commun. 2022 Feb 16;13:906. doi: 10.1038/s41467-022-28542-y (PMC8850425; doi:10.1038/s41467-022-28542-y)
Supplement: Supplementary file 1 — Supplementary information [file 41467_2022_28542_MOESM1_ESM.pdf]

Supplementary Information

Large-gap insulating dimer ground state in monolayer $IrTe_2$

Jinwoong Hwang[#*], Kyoo Kim[#], Canxun Zhang[#], Tiancong Zhu, Charlotte Herbig, Sooran Kim, Bongjae Kim, Yong Zhong, Mohamed Salah, Mohamed M. El-Desoky, Choongyu Hwang, Zhi-Xun Shen, Michael F. Crommie & Sung-Kwan Mo[*]

# These authors contributed equally

* Corresponding authors: jinwoonghwang@lbl.gov, skmo@lbl.gov

Table of Contents

Supplementary Note 1: Determination of the lattice parameters of ML $IrTe_2$

Supplementary Note 2: Structural properties of bulk $IrTe_2$

Supplementary Note 3: Characterization of BL $IrTe_2$

Supplementary Note 4: STM topograph and STS gap determination of ML $IrTe_2$

Supplementary Note 5: Temperature-dependence of ML $IrTe_2$

Supplementary Note 6: Substrate effect

Supplementary Note 7: Details of the DFT calculation for ML $IrTe_2$

Supplementary Note 8: Calculated electronic structures of ML and BL 1*T*-$IrTe_2$

Supplementary Note 9: Effect of SOC and on-site U on the electronic structure of ML $IrTe_2$

Supplementary Note 10: Analysis of lowest unoccupied molecular orbital in ML $IrTe_2$

Supplementary Note 11: Polarization dependent ARPES measurements of ML $IrTe_2$

Supplementary Note 1: Determination of the lattice parameters of ML $IrTe_2$

To more clearly see the formation of the 2 × 1 structure and determine the lattice parameters for ML $IrTe_2$, here we show a fast Fourier transformation (FFT) (Supplementary Fig. 1) from the STM topograph in Fig. 1**e**. From the position of the Bragg peaks, we deduce the primary reciprocal vectors to be $\frac{G_1}{2\pi}$ = 1.56 nm^{-1}, $\frac{G_2}{2\pi}$ = 2.57 nm^{-1}, and the angle between them to be θ = 85˚. The ratio between the principle reciprocal vectors and the angle between them is clearly different from the FFT-STM from the undistorted hexagonal 1T phase of other TMDs. This provides indisputable evidence of the formation of the distorted 2 × 1 structure. The corresponding real-space lattice parameters are a = 6.4 Å, b = = 3.9 Å and γ = 85 ˚. We then repeat the above procedure for other ML areas and obtain the average values a = 6.28 ± 0.06 Å, b = 3.92 ± 0.06 Å, γ = 85 ± 2° as reported in the main text.

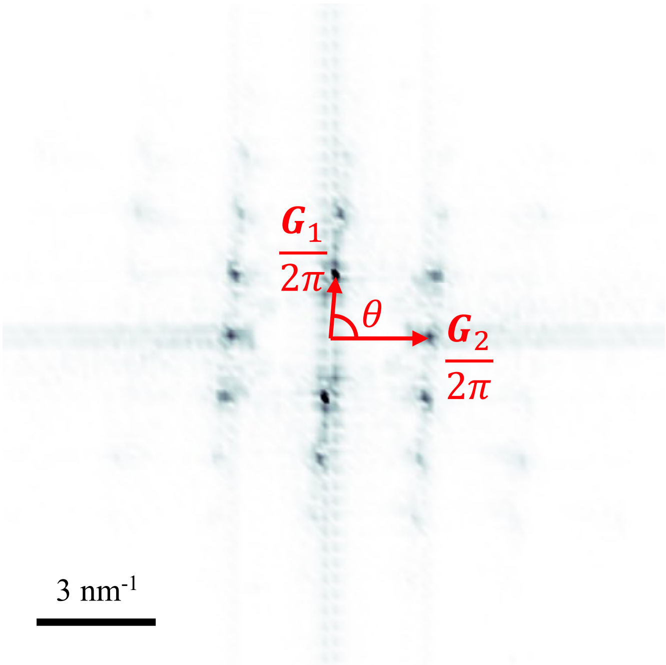

Supplementary Figure 1 | Determination of the lattice constant of ML $IrTe_2$. FFT of the STM topograph of ML $IrTe_2$ shown in Fig. 1**e**. Red arrows represent the primary reciprocal vectors.

Supplementary Note 2: Structural properties of bulk $IrTe_2$

Among the family of TMDs, 1*T*-$IrTe_2$ is unique since Te-Te interlayer coupling possesses a more covalent nature, resulting in a shorter interlayer distance than what is expected for a typical van der Waals material[1-4]. The shorter bonding distance between $IrTe_2$ layers induces the formation of a polymeric $(Te_2)^{3-}$ and stabilizes the trigonal structure ($P\bar{3}m1$) with effective Ir^{3+} valence state[2-4] as shown in Supplementary Fig. 2**a**. Since the structural transitions in $IrTe_2$ also involve Ir 5*d* to Te 5*p* charge transfer with the Te-Te bond breaking[1,2], the $IrTe_2$ layer responds to the interlayer coupling more sensitively than other conventional van der Waals layered materials[1]. When the Te-Te bonds are partially lost, e.g., by cooling in the bulk $IrTe_2$ case, the system undergoes a first-order structural transition[2-4]. The resulting triclinic structure ($P1$), which remains metallic, shows Ir^{3+}-Ir^{4+} charge-ordered stripe phases forming Ir^{4+} dimers (Supplementary Fig. 2**b**) with superstructural modulation $5 \times 1 \times 5$ at ~280 K and $8 \times 1 \times 8$ at ~180 K, as well as 6×1 at the surface[2-4].

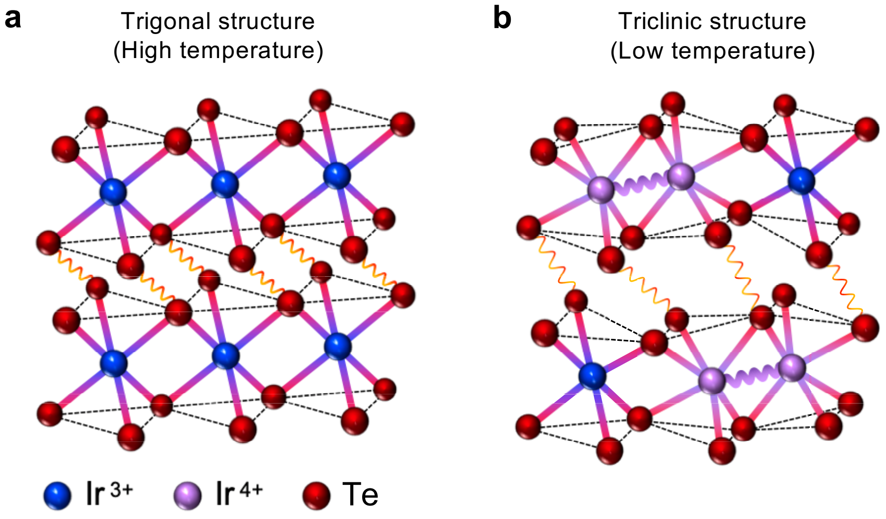

Supplementary Figure 2 | Crystal structures of bulk $IrTe_2$. a,b, Schematics of the crystal structure of bulk $IrTe_2$ for **a**, trigonal structure and **b**, triclinic structure with Ir dimers. The orange and purple wavy lines indicate the polymeric Te-Te bonding and Ir-Ir dimerization, respectively.

Supplementary Note 3: Characterization of BL $IrTe_2$

We have performed STM/STS measurements on BL $IrTe_2$. BL $IrTe_2$ mainly shows 3×1 periodicity (Supplementary Fig. 3**b**), but 5×1 periodicity is also observed in some regions of samples (Supplementary Fig. 3**c**). Although other periodicities such as $(5 + 3) \times 1$ and $(3 + 3) \times 1$ are possible in BL due to the similar formation energy on the surface[1-4], they are not clearly observed in our measurements. Most importantly, the 2×1 periodicity of ML is not found in BL $IrTe_2$. This is clearly distinguished by the STS dI/dV measurements since the 2×1 structure shows a large band gap (Fig. 2 & Supplementary Fig. 5) while BL $IrTe_2$ always exhibits metallicity, even for positions corresponding to the Ir dimers (Supplementary Figs. 3**c**,**d**). ARPES and Ir 4*f* core level measurements of BL $IrTe_2$ support the STM/STS results. ARPES intensity maps of BL $IrTe_2$ show metallic dispersion crossing the Fermi energy, and the X-shaped features found in ML (Fig. 2) completely disappears in binding energies ~1.5 – 4 eV (Supplementary Figs. 4**a-d**), indicating the absence of insulating 2×1 structure in BL. In addition, Ir 4*f* valence state of BL exhibits a clear shift to lower binding energy (~0.2 eV) compared to ML, which is caused by less screening of nuclear potential due to fewer valence electrons in Ir^{3+} than in Ir^{4+} by the polymeric Te-Te interlayer coupling (Supplementary Fig. 4**e**)[1-4]. BL Ir 4*f* valence peak is located between Ir^{4+} and Ir^{3+} states, indicating the Ir^{3+}-Ir^{4+} mixed-valence state in BL analogous to the surface of bulk $IrTe_2$[2,4].

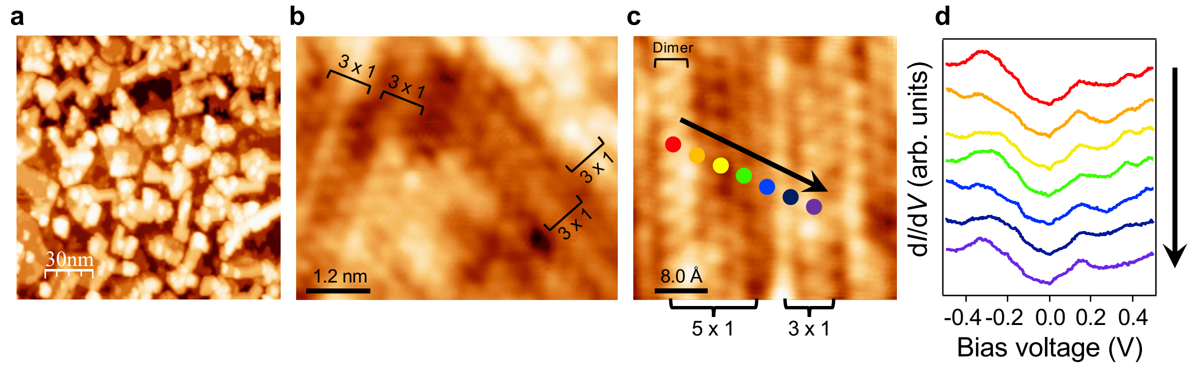

Supplementary Figure 3 | Atomic structure and STS dI/dV of BL $IrTe_2$. a, Large-scale STM topographic image of mostly BL $IrTe_2$ on BLG substrate (V_s = 1.5 V, I_0 = 0.01 nA, T = 4.7 K). **b,c**, STM topographic images of BL $IrTe_2$ film on BLG substrate (V_s = 10 mV, I_0 = 1 nA, T = 4.7 K). BL $IrTe_2$ mainly shows **b**, 3 × 1 and **c**, 5 × 1 atomic structures. **d,** STS dI/dV spectra obtained at the denoted positions in Supplementary Fig. 3**c** (modulation voltage V_{rms} = 5 mV).

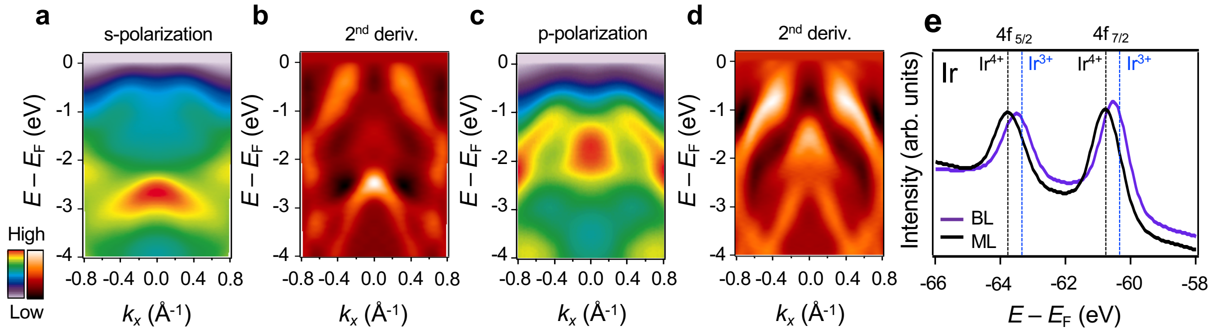

Supplementary Figure 4 | ARPES intensity maps and Ir 4*f* core levels of BL $IrTe_2$. a, ARPES intensity maps of BL $IrTe_2$ taken along the M–Γ–M direction using *s*-polarized photons (T = 13 K). **b**, Its second derivative with respect to momentum. **c**, Same as **a**, but acquired using *p*-polarized photons. **d**, Its second derivative with respect to momentum. **e**, Comparison of Ir 4*f* core-level spectra between ML and BL $IrTe_2$. Black and blue dashed lines indicate Ir^{4+} and Ir^{3+} valence state energies[2], respectively.

Supplementary Note 4: STM topograph and STS gap determination of ML $IrTe_2$

While BL $IrTe_2$ shows various periodicities (Supplementary Fig. 3) like the surface of bulk $IrTe_2$, ML $IrTe_2$ only shows the 2×1 Ir dimerized structure (Supplementary Fig. 5**a**). Domains are found to be rotated by 120° with respect to each other (Supplementary Figs. 3**b** and 5**a**). This type of energetically equivalent 120°-rotated domains is common for distorted 1*T*-TMDs grown on BLG substrate[5-8]. Some domains show more distortion due to the strain or defects[5,8].

In our STS d*I*/d*V* measurements on ML $IrTe_2$, no in-gap state has been observed at the interface between different rotational domains. A small (~0.05 eV) rigid shift of the whole spectrum, due to the local electrostatic environment, is observed depending on the measurement position (Supplementary Figs. 5**a**,**b**). However, domain size or the absolute energy position of the onset of the gap does not affect the size of the energy gap (E_g) significantly (Supplementary Table 1). The STS gap value of 1.02 ± 0.05 eV was determined through a statistical analysis of 73 d*I*/d*V* spectra (Supplementary Fig. 5**c**).

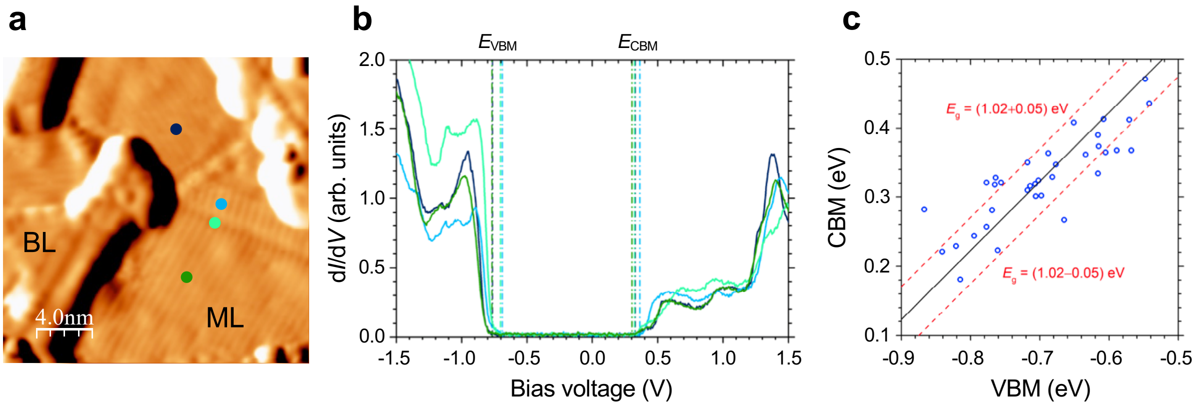

Supplementary Figure 5 | STS gap determination of ML $IrTe_2$. a, A first derivative STM topography image of ML $IrTe_2$ (V_s = 1.5 V, I_0 = 0.1 nA, T = 4.7 K). **b**, ML STS dI/dV spectra obtained at the denoted positions in Supplementary Fig. 5**a** (modulation voltage V_{mod} =10 mV). **c**, The STS gap determination from the statistical analysis of dI/dV.

Supplementary Table 1 | Summary of averaged E_g in ML $IrTe_2$ with respect to its domain size.

**Domain size (nm)**	**Averaged E_g (eV)**
4.5 ~ 9.5	1.03 ± 0.03
9.5 ~ 13.5	1.04 ± 0.06
13.5 ~ 18.5	1.00 ± 0.05

Supplementary Note 5: Temperature-dependence of ML $IrTe_2$

We have performed the temperature-dependent ARPES and core levels measurements for ML $IrTe_2$. We found that the size of the gap and ARPES band dispersion of ML does not change up to 300 K (Supplementary Figs. 6**a-c**). The temperature-dependent angle-integrated spectra of the valence band and core level spectra for Ir 4*f* and Te 4*d* do not show any peak shift neither (Supplementary Figs. 6**c-e**). These are distinct from what has been observed from bulk $IrTe_2$ and its surface[1-4], which show multiple transitions of Ir valence state and valence band spectra. It is also completely different from thicker (~80 nm) films of $IrTe_2$[9], in which suppression of the dimer structure was found. Our findings indicate that the 2 × 1 insulating dimer structure is unique in ML, and it is robust against thermal fluctuation up to 300 K.

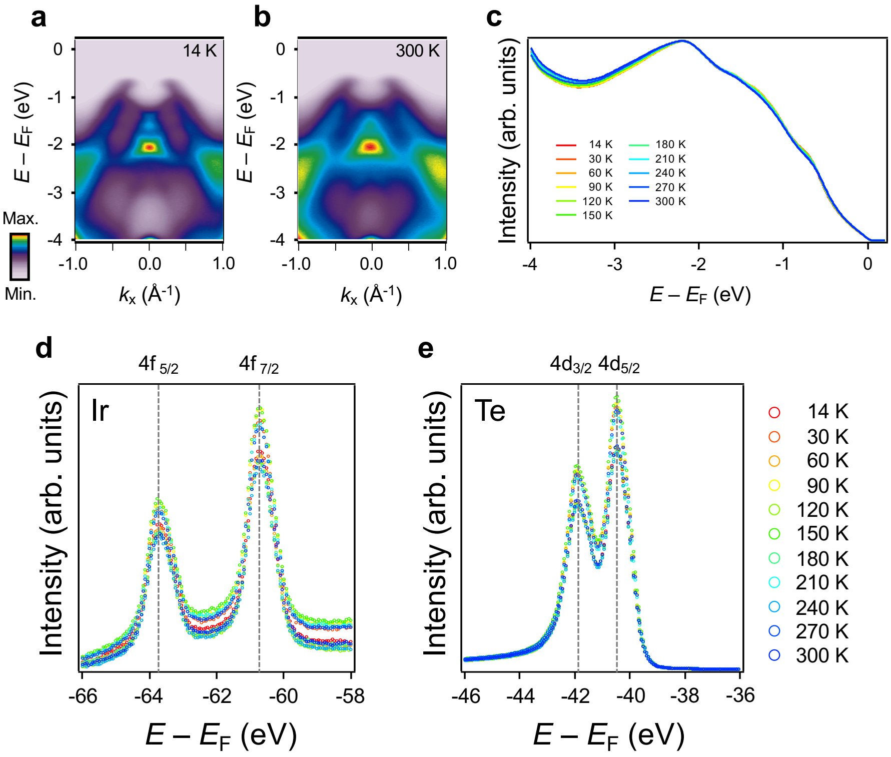

Supplementary Figure 6 | Temperature-dependent ARPES results and core levels of ML $IrTe_2$. a,**b**, ARPES intensity maps of ML $IrTe_2$ acquired at **a**, 14 K and **b**, 300 K using *p*-polarized photons. **c**, Temperature-dependent angle-integrated valence band ARPES spectra. **d**,**e**, Temperature-dependent core levels of **d**, Ir 4*f* and **e**, Te 4*d* states.

Supplementary Note 6: Substrate effect

The influence from the underlying bilayer graphene (BLG) substrate should be carefully investigated because the substrates can, in general, modify the physical properties of thin films and sometimes very strongly. When the interaction between BLG and the overlaid materials is strong, the evidence of the effect is also observed in BLG π band because the interaction is mutual as a result of coupling between the wavefunctions of BLG substrate and overlaid materials. The well-known examples of such changes in the electronic structure of BLG stacked with other materials include the formation of hybridization gap[10], segmentation of the BLG π bands[11], changes in band velocity[12,13], kink in the BLG bands[14], and so on. As shown in Supplementary Figs. 7**a-d**, however, the BLG π bands from ML $IrTe_2$/BLG structure do not show any relevant changes.

Moreover, the interface between $IrTe_2$ film and BLG is incommensurate, as shown in Supplementary Fig. 7**e**. ML $IrTe_2$ is rotated to ~10° from the BLG (Supplementary Fig. 7**e**). This incommensuracy makes the potential felt by the $IrTe_2$ from BLG essentially random perturbations rather than a coherent superpotential. The net effect is similar to impurity scattering, resulting in broader RHEED lines (Fig. 1**b**) compared to those from TMDs that are grown with a well-aligned azimuthal angle[15-17]. Hence, the effect from the BLG substrate to the low-energy electronic structure of $IrTe_2$ film is minimal or even negligible.

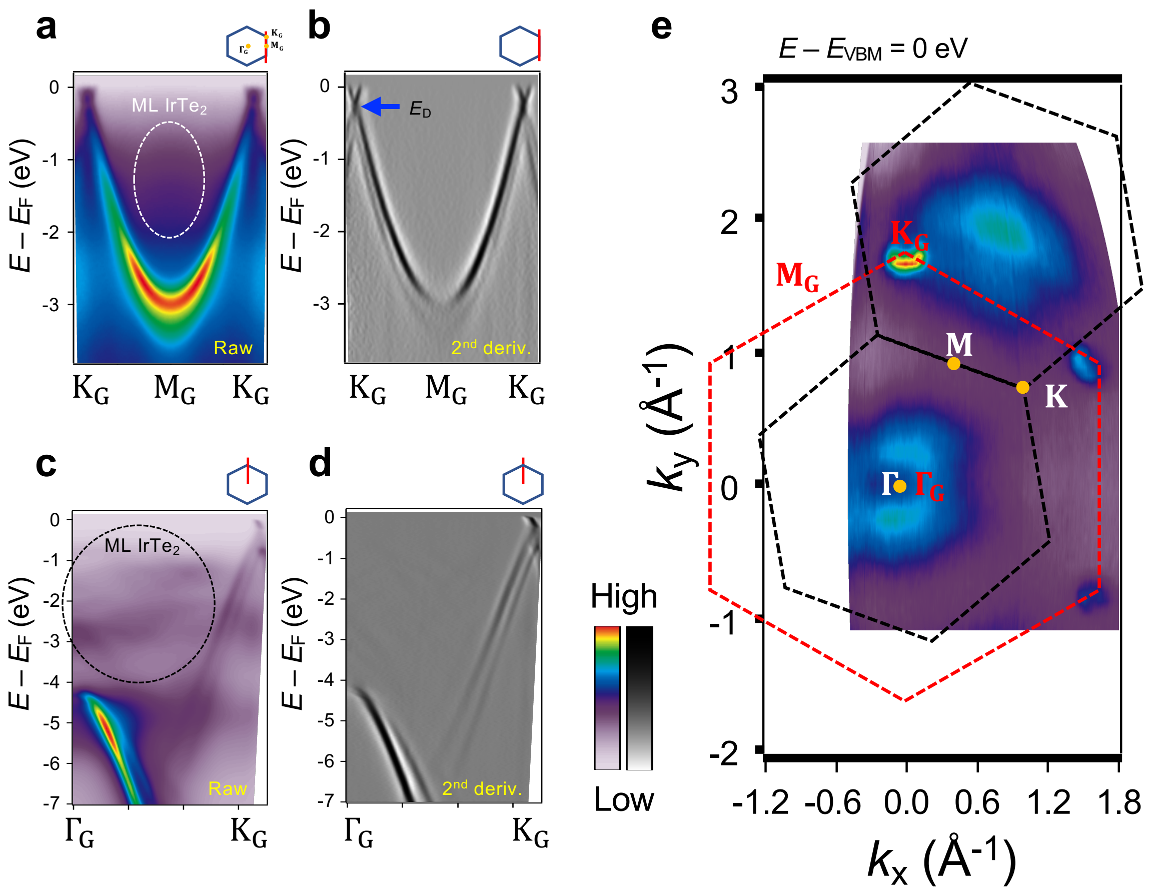

Supplementary Figure 7 | ARPES spectra of BLG π bands. a-d, ARPES intensity maps taken along **a**,**b**, K$_G$–M$_G$–K$_G$ and **c**,**d**, Γ$_G$–K$_G$ directions of Brillouin zone of BLG and their second derivatives, respectively. **e**, Constant energy map obtained from $E - E_{VBM} = 0$ eV. Red and black dashed hexagons represent the Brillouin zones of BLG and averaged three domains of 2 × 1 $IrTe_2$, which are described in Supplementary Note 7, respectively.

Supplementary Note 7: Details of the DFT calculation for ML $IrTe_2$

Regarding the three different rotational domains (see Supplementary Note 4), there can be three soft q vectors (q = -q assumed) in high symmetry ML $IrTe_2$ with hexagonal CdI2 type structure, which gives rise to six low symmetry structures. To compare the ARPES spectra with DFT calculation, we unfolded the band structure of 2 × 1 ML $IrTe_2$ with different q vectors (Supplementary Fig. 8**a**) into 1 × 1 distorted hexagonal Brillouin zone (BZ) (Supplementary Fig. 8**b**) of the hypothetical high symmetry phase of ML $IrTe_2$. In the unfolding procedure, we first picked a single direction, say M2, and averaged the unfolding spectra for each domain with different periodicities, as shown in Supplementary Fig. 8**a**. The results are shown in Supplementary Figs. 8**c**-**d**. The domain averaging induces the complex X-shape band features observed in ARPES. A slight difference at the zone boundary from the different BZ is seen but is negligible.

Moreover, we have also calculated an artificial 5 × 1 structure (Supplementary Fig. 9) as an anti-example of the insulating electronic structure of ML 2 × 1 structure. Not only 5 × 1 has higher energy than 2 × 1 by ~0.230 eV per formula unit, but also the band structure of 5 × 1 structure clearly shows a metallic behavior. The comparison represents that 2 × 1 insulating dimer ground state in ML is unique, unlike the case of other structural candidates 5 × 1, 8 × 1… etc., which were observed in bulk and surface $IrTe_2$[1-4].

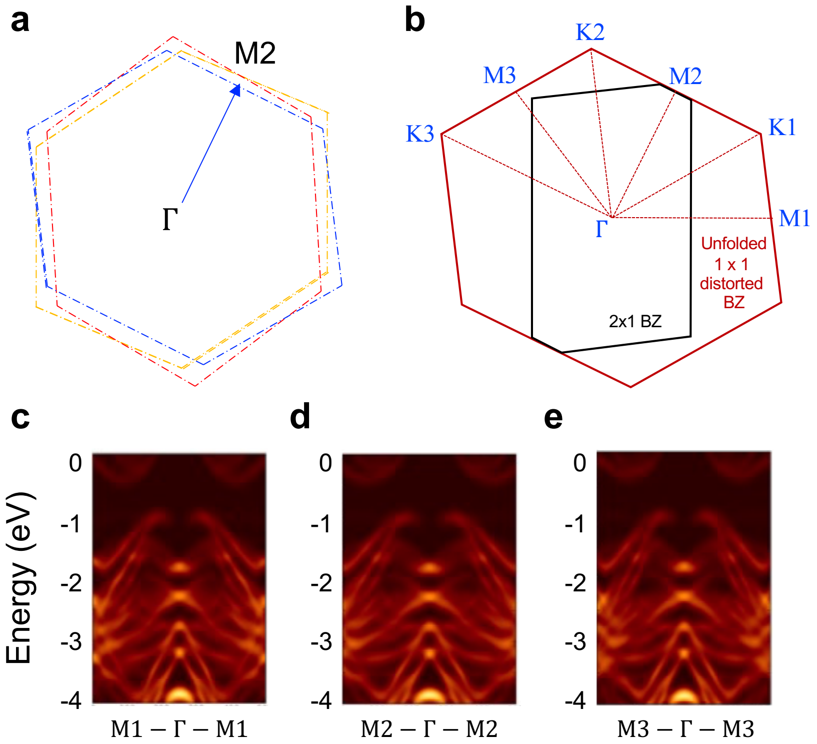

Supplementary Figure 8 | Description for DFT calculation of ML 2 × 1 $IrTe_2$. a, The relation between the BZs for three different domains. **b**, The relation between an unfolded 1 × 1 distorted hexagonal (Red) and the distorted 2 × 1 (Black) BZs. **c-e**, Calculated domain averaged DFT spectral weights along **c**, M1–Γ–M1, **d**, M2–Γ–M2, and **e**, M3–Γ–M3, respectively.

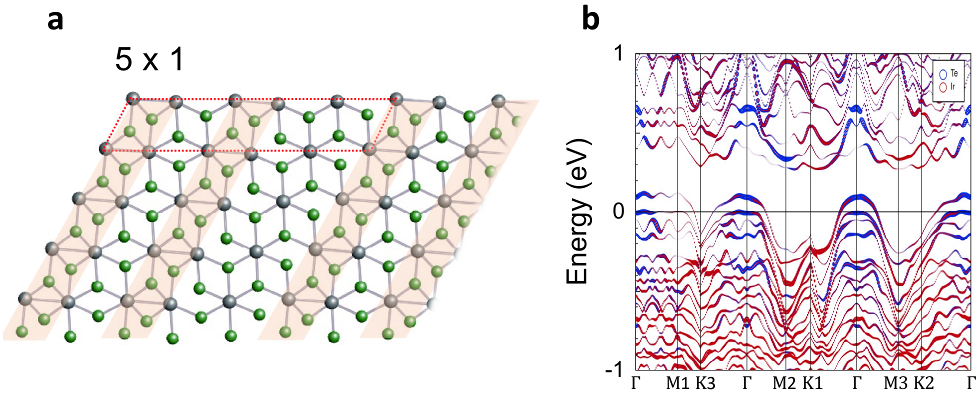

Supplementary Figure 9 | Calculation of artificial 5 × 1 ML 1*T*-$IrTe_2$. a, Relaxed structure in 5 × 1 supercell. All the Ir forms dimer except one, which is restricted by choice of supercell. **b**, Unfolded band structure for the 5 × 1 structure.

Supplementary Note 8: Calculated electronic structures of ML and BL 1*T*-$IrTe_2$

Supplementary Figure 10**a** presents the calculated band structure of undistorted ML 1*T*-$IrTe_2$. The DFT band structure is entirely different from the measured band structure of the 2 × 1 dimer phase (Fig. 3). The Fermi surface (FS) topology of ML 1*T*-$IrTe_2$ (Supplementary Fig. 10**c**) is reminiscent of the hidden FS nesting feature in low-dimensional CDW systems[18]. The calculated imaginary part of the electronic susceptibility, which corresponds to the nesting intensity, also shows a strong peak structure at q = **M** (Supplementary Fig. 10**d**). Well-defined FS nesting condition, $\vec{q}_{\mathrm{CDW}} = \overrightarrow{\mathrm{M}}$, is indeed obtained in ML 1*T*-$IrTe_2$ (Supplementary Fig. 10**g**) with significant contribution from Te orbitals (Supplementary Fig. 10**a**).

Supplementary Figures 10**b** and 10**e** show the DFT band structure and Fermi surface of undistorted BL 1*T*-$IrTe_2$, respectively. Compared to ML (Supplementary Fig. 10**a**), BL 1*T*-$IrTe_2$ shows multiple band crossings at Fermi energy due to the band and Fermi surface splitting (Supplementary Figs. 10**b**,**e**) by the recovered Te-Te interlayer coupling. The split Fermi surface of BL weakens the FS nesting condition, resulting in a suppression of the charge instability at the M-point (Supplementary Fig. 10**f**). Although the weak feature of charge susceptibility is obtained at $\vec{q} = \overrightarrow{\mathrm{M}}/4$, the calculated phonon dispersion of BL 1*T*-$IrTe_2$ does not show soft phonon along the high symmetry directions (Supplementary Figs. 10**h**).

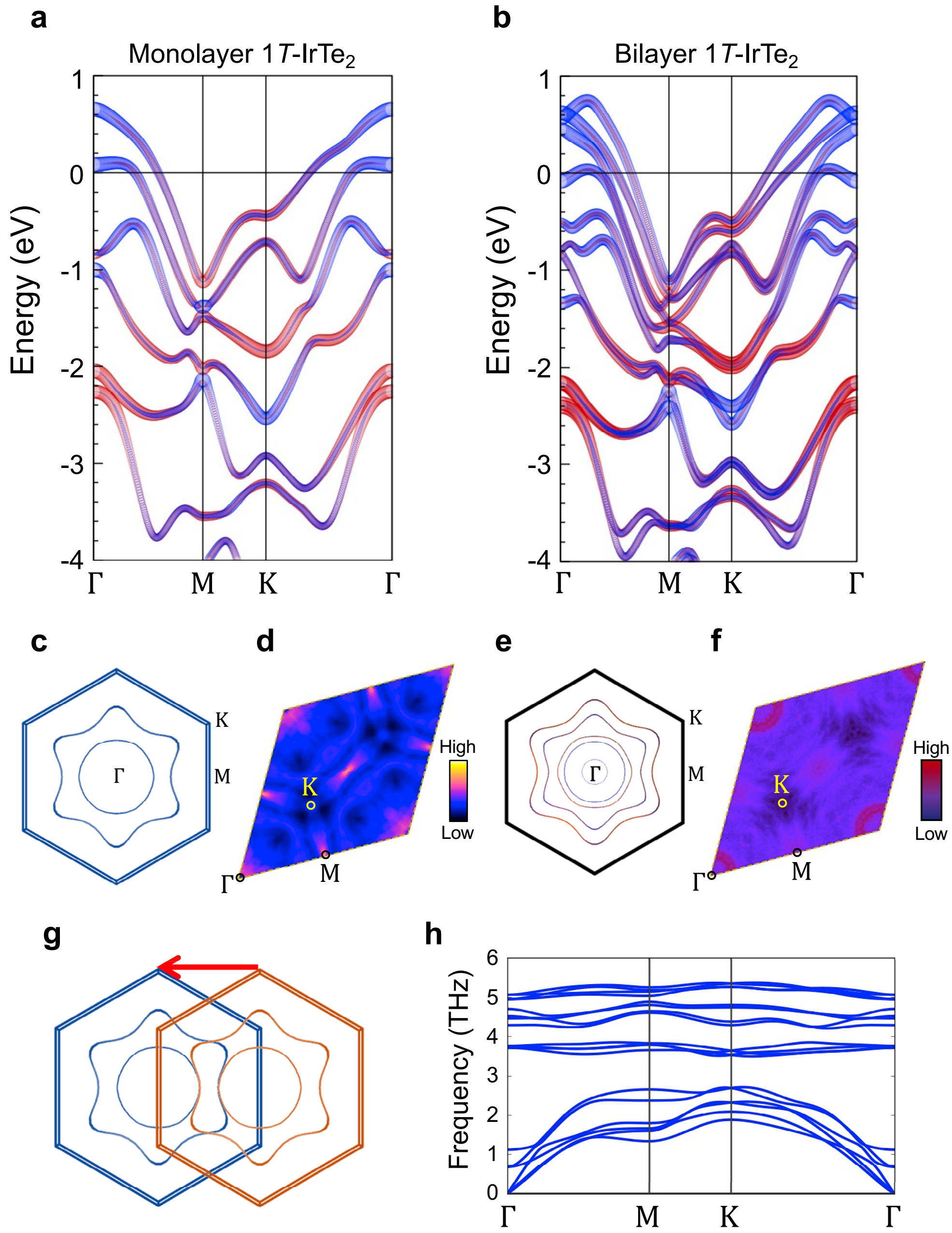

Supplementary Figure 10 | Calculated electronic and phonon properties of undistorted ML and BL 1*T*-IrTe₂. a,**b**, Calculated DFT band structure of **a**, ML and **b**, BL 1*T*-$IrTe_2$. Blue and red colors represent Te and Ir contributions, respectively. **c-f**, Calculated **c**,**e**, Fermi surface and **d**,**f**, the imaginary part of the electronic susceptibility Im $\chi(\boldsymbol{q})$ of ML and BL 1*T*-$IrTe_2$, respectively. The outer hexagon represents the Brillouin zone of 1*T*-$IrTe_2$. **g**, Description of the Fermi surface nesting in ML 1*T*-$IrTe_2$ corresponding to $\vec{q}_{CDW} = \vec{M}$ (red arrow). **h**, Calculated phonon dispersion of BL 1*T*-$IrTe_2$.

Supplementary Note 9: Effect of SOC and on-site *U* on the electronic structure of ML $IrTe_2$

To theoretically investigate the large gap size in ML $IrTe_2$, we have performed DFT calculations including spin-orbit coupling (SOC) and on-site Coulomb interaction *U*. The effect of SOC in the 2 × 1 dimer structure is negligible (Supplementary Fig. 11). Moreover, the on-site *U* not only decreases the gap, but also induces a metallic state for high values of *U* larger than 3 eV (Supplementary Fig. 12). GW_0 calculation, which includes the non-local screening, gives a reasonable result, despite the overestimated gap (~1.7 eV) compared to the STS d*I*/d*V* gap (~1.02 ± 0.05 eV). This is due to that GW_0 calculation does not fully account for the grey shadow range of the STS d*I*/d*V* spectrum (Supplementary Fig. 13). However, the density of states profile from GW_0 calculation becomes more in agreement with the STS d*I*/d*V* spectrum in other energy ranges compared to that from DFT (Supplementary Fig. 13). This result indicates that the non-local screening plays an important role in understanding the large gap of ML $IrTe_2$.

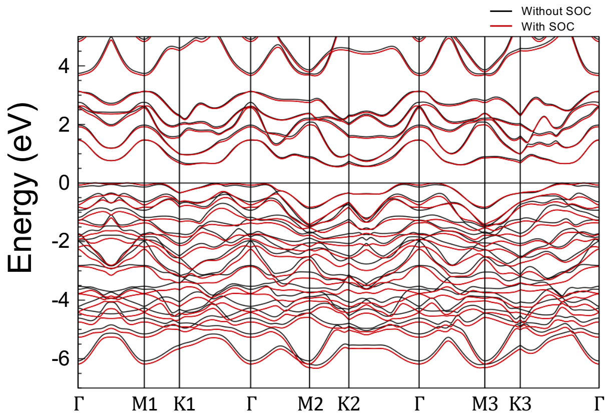

Supplementary Figure 11 | Effect of the spin-orbital coupling (SOC) on ML 2 × 1 $IrTe_2$. Comparison between calculated DFT band structures without (black) and with (red) SOC for the 2 × 1 dimer structure of ML $IrTe_2$ unfolded into the hexagonal Brillouin zone (Supplementary Fig. 8**b**).

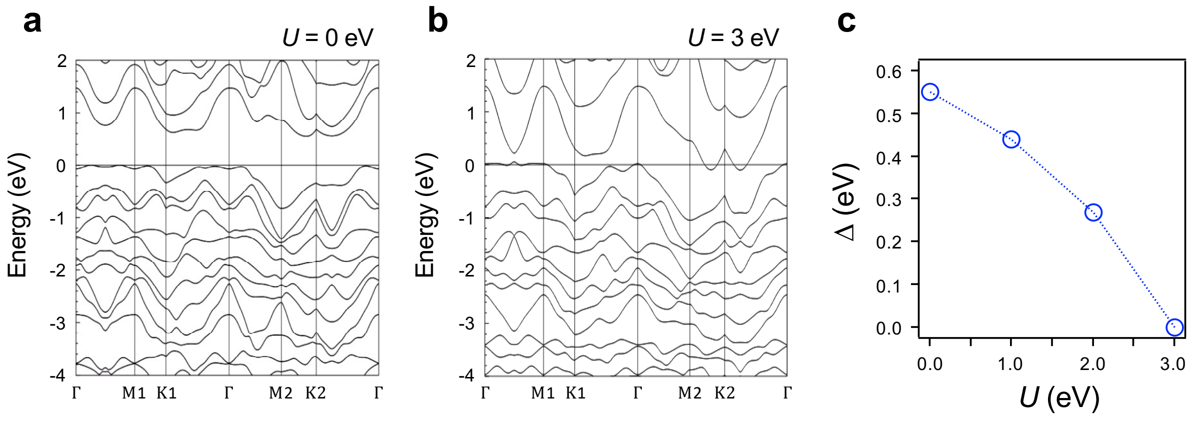

Supplementary Figure 12 | Effect of the on-site Coulomb interaction *U* on ML $IrTe_2$. a,b, The DFT band structures with **a**, $U = 0$ eV and **b**, $U = 3$ eV for the 2 × 1 dimer structure of ML $IrTe_2$. **c**, The electronic band gap size Δ with respect to *U*.

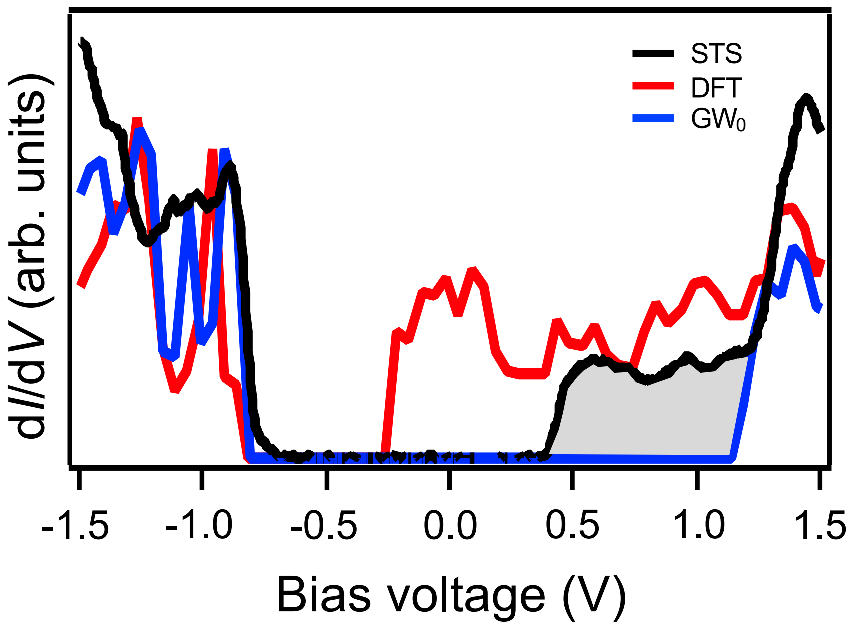

Supplementary Figure 13 | Comparison of the density of states profiles for the 2 × 1 dimer structure of ML $IrTe_2$ obtained from STS (black), DFT (red), and GW_0 (blue). The grey shadow area indicates the energy range in which the STS d*I*/d*V* is not captured by the GW_0 result.

Supplementary Note 10: Analysis of lowest unoccupied molecular orbital in ML $IrTe_2$

Supplementary Figure 14 presents the orbital character of the lowest unoccupied molecular orbital (LUMO) of ML $IrTe_2$. It consists of antibonding states of d_{xy} orbitals of dimerized Ir atoms (Supplementary Fig. 14**a**), and $p_{x'}$ orbitals of neighboring Te1 atoms (Supplementary Fig. 14**b**), as in bulk $IrTe_2$. Local axes for these orbitals are slightly different, which might come from the Ir-Te2 interaction. The wave function of the LUMO at Γ-point is well localized to Ir-Te1-Te1-Ir stripes, as shown in Supplementary Fig. 14**c**. The outer Ir d_{xy} orbitals in the stripe also have mild interaction with Te2 p orbitals which gives a complicated projection profile of band structure, as shown in Supplementary Fig. 14**a**.

On the other hand, the bonding state of Ir dimer and neighboring Te1 atoms is strongly hybridized with Te2 orbitals, and the orbital projection is eventually distributed over a large energy window (not shown here). Supplementary Figure 14**d** presents the projected densities of states to Ir $5d_{xy}$ and Te1 $5p_{x'}$. The LUMO state shown in Supplementary Figs. 14**c**,**d** is basically identical to those in previous literature except that the contribution of Te1 orbitals is much stronger for ML case than that of bulk $IrTe_2$[19, 20]. This strong involvement of Te1 $5p_{x'}$ orbital can be the origin of 2nd order structural transition in ML $IrTe_2$. This is represented by the phonon instability in Fig. 4**c,** which is not observed in bulk case[21]. Delicate balancing between local Ir dimer and Te itinerant physics explains the different behavior in structural transition and/or phonon soft mode between bulk and ML $IrTe_2$. Saleh et al. also conducted DFT calculations for the 5×1 structure of monolayer[20], which stabilizes over the 1×1 structure. However, when we compare its energy to that of the 2×1 structure, the 5×1 structure is energetically less stable due to the undimerized pairs of Ir atoms. This suggests that the main source of energy gain is Ir dimerization, while the Te band is responsible for the instability of the 1×1 structure and initiates the structural transition to the 2×1 structure.

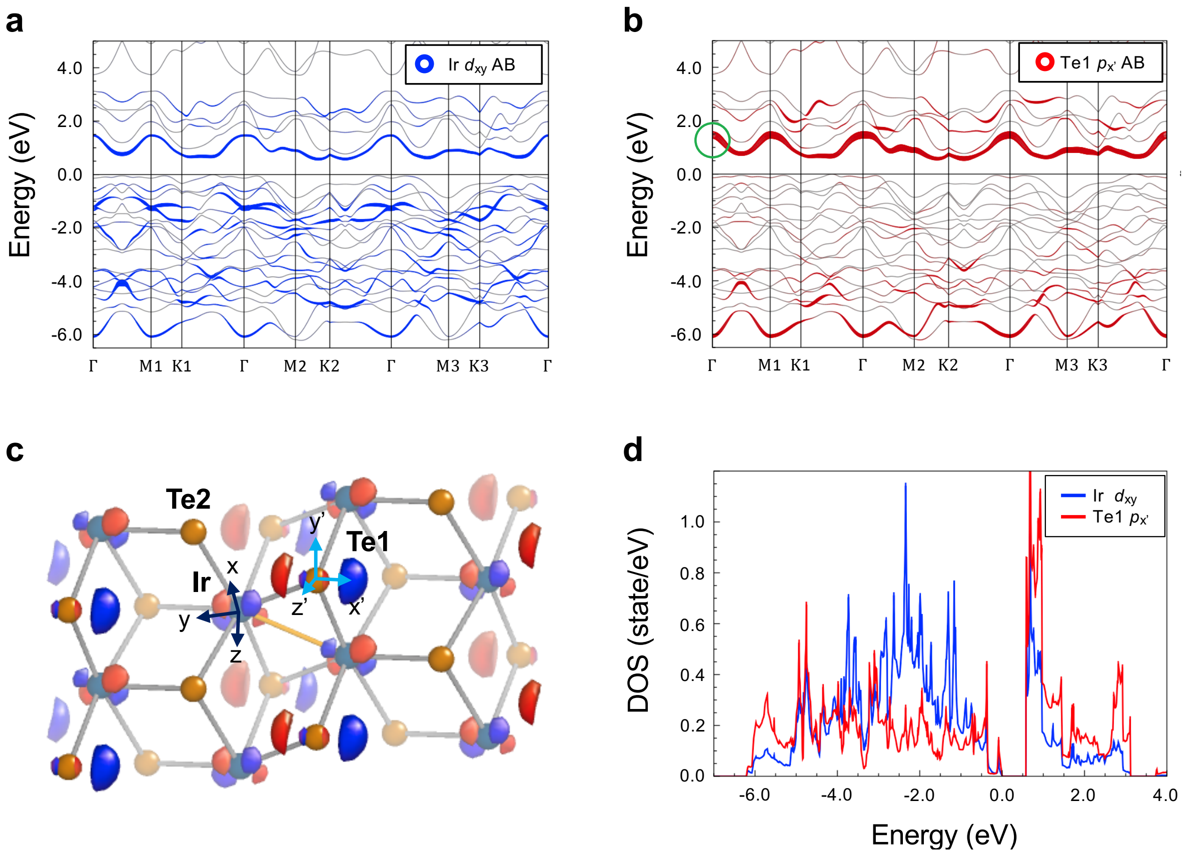

Supplementary Figure 14 | Lowest unoccupied molecular orbital (LUMO) of $IrTe_2$ monolayer. **a**,**b**, Band projection to **a**, Ir 5d_{xy} and **b**, Te1 5$p_{x'}$ antibonding states. **c**, Wave function of the LUMO state marked by green circle at Γ-point of Supplementary Fig. 14**b**. The local axes for orbital projection are depicted with black and cyan arrows for Ir and Te1, respectively. **d**, Projected density of states to orbitals of Ir 5d_{xy} and Te1 5$p_{x'}$. The spin-orbit interaction is turned off to remove complex phase term for visualization purposes, which does not change the discussion much.

Supplementary Note 11: Polarization dependent ARPES measurements of ML $IrTe_2$

The changes in the photocurrent intensity for the two different polarizations are primarily originated from the matrix element effects during the photoemission process, which encode the orbital character of the bands. The matrix element of a photoemission process can be described as $\left|M_{f,i}^{k}\right|^2 \propto \left|\langle\psi_f^k|\hat{\varepsilon}\cdot\boldsymbol{r}|\psi_i^k\rangle\right|^2$, where $\hat{\varepsilon}$ is the unit vector of the electric field of the light, ψ_f^k and ψ_i^k are the final and initial state wave functions of the photoelectrons, and $\boldsymbol{r}$ is the position of the electrons. Within the dipole approximation, the electronic state in the crystal needs to be odd (even) with respect to the reflection in the scattering plane in order to lead to a non-zero photocurrent for *s*- (*p*-) polarized light. Within an experimental configuration sketched in Supplementary Fig. 15**a**, where both the analyzer slit and incident light are in the mirror plane defined by the analyzer slit and the sample surface normal, *p*- (electric field vector in the mirror plane) and *s*- (electric field vector perpendicular to the mirror plane) polarized photons make $\hat{\varepsilon}\cdot\boldsymbol{r}$ even and odd, respectively.

For the incoming p-polarized photons, the photocurrents from $(d_{z^2}, d_{xz}, d_{x^2-y^2})$-orbitals are enhanced because these orbitals are even with respect to the mirror plane, while the others are dominant in the measurements with *s*-polarized light. However, in the case of ML $IrTe_2$, due to the three equivalent rotational domains shown in Supplementary Fig. 5, it is difficult to relate all the orbitals to the scattering plane. Only one of them, d_{z^2}, is perpendicular to the layer plane, and it gets constantly enhanced by the p-polarized photons even in the presence of the azimuthal misorientation (Supplementary Fig. 15**b**,**c**). Hence, ARPES band structures in Fig. 3 obtained with p-polarized light corresponds well to the calculated band projection of d_{z^2} orbital, while the other orbitals are represented well by the data measured with *s*-polarized light, considering the existence of three equivalent rotational domains.

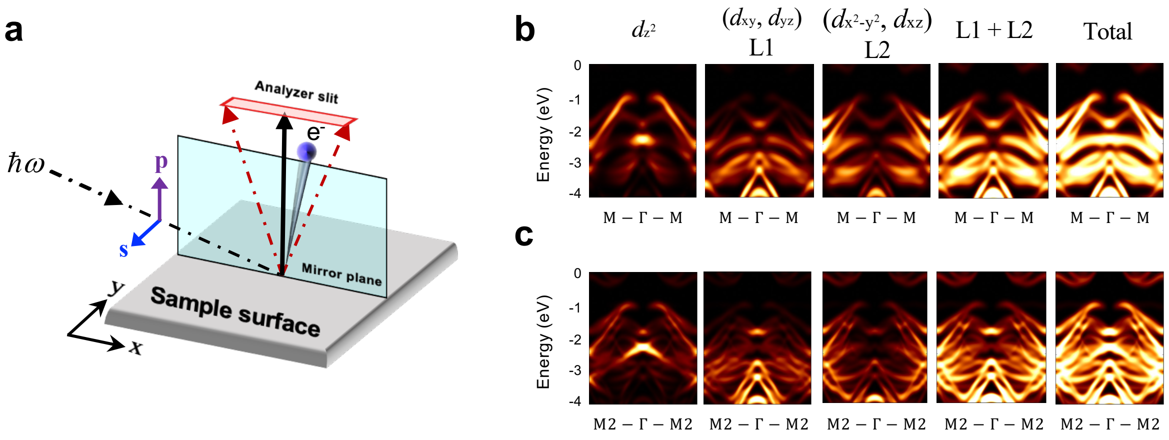

Supplementary Figure 15 | Experimental setup for ARPES measurement and DFT simulations. **a**, Schematics of the experimental setup for polarization-dependent ARPES measurement. **b**,**c**, DFT calculations of distorted 2 × 1 ML $IrTe_2$ depending on the orbital characters for **b**, single and **c**, averaged three equivalent domains, respectively.

Supplementary References

1. Kim, K., Kim, S., Ko, K.-T., Lee, H., Park, J.-H., Yang, J.-J., Cheong, S.-W. & Min, B. I. Origin of First-Order-Type Electronic and Structural Transitions in $IrTe_2$. *Phys. Rev. Lett.* **114**, 136401 (2015).

2. Ko, K.-T. *et al.* Charge-ordering cascade with spin-orbit Mott dimer states in metallic iridium ditelluride. *Nat. Commun.* **6**, 7342 (2015).

3. Ootsuki, D. *et al.* Orbital degeneracy and Peierls instability in the triangular-lattice superconductor $Ir_{1-x}Pt_xTe_2$. *Phys. Rev. B.* **86**, 014519 (2012).

4. Rumo, M. *et al.* Examining the surface phase diagram of $IrTe_2$ with photoemission. *Phys. Rev. B.* **101**, 235120 (2020).

5. Tang, S., Zhang, C., Wong, D. *et al.* Quantum spin Hall state in monolayer 1T'-WTe_2. *Nat. Phys.* **13**, 683-687 (2017).

6. Tang, S. *et al.* Electronic structure of monolayer 1T'-$MoTe_2$ grown by molecular beam epitaxy. *APL Materials* **6**, 026601 (2018).

7. Choi, B. *et al.* Visualizing Orbital Content of Electronic Bands in Anisotropic 2D semiconducting $ReSe_2$. *ACS. Nano* **14**, 7880-7891 (2020).

8. Zhao, C. *et al.* Strain Tunable Semimetal-Topological-Insulator Transition in Monolayer 1T'-WTe_2. *Phys. Rev. Lett.* **125**, 046801 (2020).

9. Yoshida, M., Kudo, K., Nohara, M. & Iwasa, Y. Metastable Superconductivity in Two-Dimensional $IrTe_2$ Crystals. *Nano Letters* **18**, 3113-3117 (2018).

10. Hwang, J. *et al.* Hole doping, hybridization gaps, and electronic correlation in graphene on a platinum substrate. *Nanoscale* **9**, 11498 (2017).

11. Diaz, H. C. *et al.* Direct Observation of Interlayer Hybridization and Dirac Relativistic Carriers in Graphene/MoS_2 van der Waals Heterostructures. *Nano Lett.* **15**, 1135-1140 (2015).

12. Hwang, C. *et al*, Fermi velocity engineering in graphene by substrate modification. *Sci. Rep*. **2**, 590 (2012).

13. Ryu, H. *et al.* Temperature-Dependent Electron-Electron Interaction in Graphene on $SrTiO_3$. *Nano Lett.* **17**, 5914-5918 (2017).

14. Hwang, J. *et al.* Tunable Kondo resonance at a pristine two-dimensional Dirac semimetal on a Kondo insulator. *Nano Lett.* **20**, 7973-7979 (2020).

15. Zhang, Y. *et al.* Direct observation of the transition from indirect to direct bandgap in atomically thin epitaxial $MoSe_2$. *Nat. Nanotech.* **9**, 111-115 (2014).

16. Zhang, Y. *et al*. Electronic Structure, Surface Doping, and Optical Response in Epitaxial WSe_2 Thin Films. *Nano. Lett.* **16**, 2485-2491 (2016).

17. Ryu, H. *et al*. Persistent charge-density-wave order in single-layer $TaSe_2$. *Nano Lett.* **18**, 689 (2018).

18. Whangbo, M. H., Canadell, E., Foury, P. & Pouget, J. P. Hidden Fermi surface nesting and charge density wave instability in low-dimensional metals. *Science* **252**, 96-98 (1991).

19. Pascut, G. L. *et al*. Dimerization-Induced Cross-Layer Quasi-Two-Dimensionality in Metallic $IrTe_2$. *Phys. Rev. Lett.* **112**, 086402 (2014).

20. Saleh, G. *et al*. First-Principles Theory of Phase Transitions in $IrTe_2$. *J. Phys. Chem. Lett.* **11**, 2127-2132 (2020).

21. Cao, H. *et al*. Origin of the phase transition in $IrTe_2$: Structural modulation and local bonding instability. *Phys. Rev. B* **88**, 115122 (2013).
